# On the origin of crossover interference: A chromosome oscillatory movement (COM) model

**DOI:** 10.1186/1755-8166-4-10

**Published:** 2011-04-08

**Authors:** Maj A Hultén

**Affiliations:** 1 Department of Molecular Medicine and Surgery and Center for Molecular Medicine, CMM L8:02, Karolinska Institutet, Karolinska University Hospital, Solna, S-17 1 76 Stockholm, Sweden; 2 Warwick Medical School, Warwick University, Coventry CV47AL, UK

## Abstract

**Background:**

It is now nearly a century since it was first discovered that crossovers between homologous parental chromosomes, originating at the Prophase stage of Meiosis I, are not randomly placed. In fact, the number and distribution of crossovers are strictly regulated with crossovers/chiasmata formed in optimal positions along the length of individual chromosomes, facilitating regular chromosome segregation at the first meiotic division. In spite of much research addressing this question, the underlying mechanism(s) for the phenomenon called crossover/chiasma interference is/are still unknown; and this constitutes an outstanding biological enigma.

**Results:**

The Chromosome Oscillatory Movement (COM) model for crossover/chiasma interference implies that, during Prophase of Meiosis I, oscillatory movements of the telomeres (attached to the nuclear membrane) and the kinetochores (within the centromeres) create waves along the length of chromosome pairs (bivalents) so that crossing-over and chiasma formation is facilitated by the proximity of parental homologs induced at the nodal regions of the waves thus created. This model adequately explains the salient features of crossover/chiasma interference, where (1) there is normally at least one crossover/chiasma per bivalent, (2) the number is correlated to bivalent length, (3) the positions are dependent on the number per bivalent, (4) interference distances are on average longer over the centromere than along chromosome arms, and (5) there are significant changes in carriers of structural chromosome rearrangements.

**Conclusions:**

The crossover/chiasma frequency distribution in humans and mice with normal karyotypes as well as in carriers of structural chromosome rearrangements are those expected on the COM model. Further studies are underway to analyze mechanical/mathematical aspects of this model for the origin of crossover/chiasma interference, using string replicas of the homologous chromosomes at the Prophase stage of Meiosis I. The parameters to vary in this type of experiment will include: (1) the mitotic karyotype, i.e. ranked length and centromere index of the chromosomes involved, (2) the specific bivalent/multivalent length and flexibility, dependent on the way this structure is positioned within the nucleus and the size of the respective meiocyte nuclei, (3) the frequency characteristics of the oscillatory movements at respectively the telomeres and the kinetochores.

## Background

**Figure 1:**
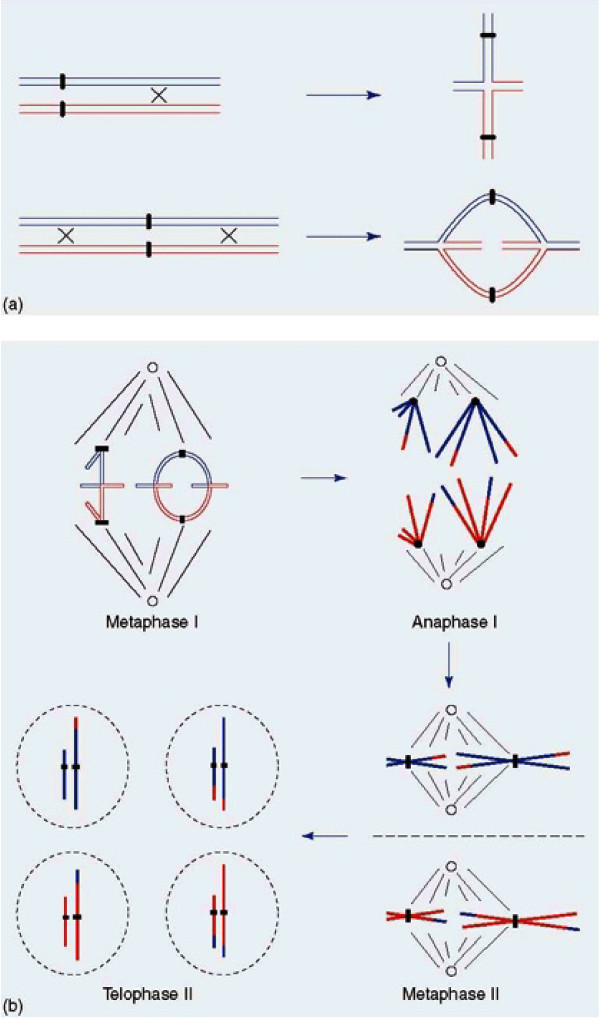
**Schematic illustration of the meiotic process**. (a) Homologous chromosome synapsis and crossing over/chiasma formation at the Pachytene stage of Prophase 1 and the derivative bivalents at the following Metaphase I. (b) Progression through Metaphase I to Anaphase I leading to the halving of the chromosome number, Metaphase II and Anaphase II where the chromatids separate (similar to mitotic Anaphase) and Telophase II comprising the four haploid daughter cell nuclei. Reproduced from [3.]

Positive crossover interference, also termed genetic or chiasma interference, i.e. the non-random placement of crossovers along the length of individual chromosomes with a reduced probability of occurrence of one crossover in the vicinity of another, is a universal feature in the outstanding majority of eukaryotic organisms. The patterns of crossovers/chiasmata on individual chromosome pairs, as governed by interference, are of crucial importance for regular segregation of the homologous parental chromosomes at the meiosis I division [review in [1-3]] as schematically illustrated in Figure 1a.

Completion of reciprocal recombination/crossing-over between parental half chromosomes (chromatids) together with chromatid cohesion, leads to the formation of chiasmata, i.e. physical connections that hold parental homologs (bivalents) together. The positional control by interference seemingly creates bivalents of optimal mechanical stability, promoting regular segregation at the subsequent Meiosis I Anaphase. This first meiotic, reductional, division leads to the chromosome number of the two daughter nuclei being halved, with the second meiotic division giving rise to haploid gametes, as illustrated in Figure 1b.

Crossover interference was first described nearly a century ago by Sturtevant and Muller in *Drosophila melanogaster *[4,5], in fact only a decade after the chromosome theory of Bovery and Sutton had been established [review in [6,7]]. Muller in his paper published in 1916 [5] wrote that "In a sense then, the occurrence of one crossing-over interferes with the coincident occurrence of another crossing-over in the same pair of chromosomes, and I have accordingly termed this phenomenon "*interference*"."

In the interim this phenomenon, that each homologous chromosome pair will receive at least one crossover/chiasma (the so-called obligate chiasma) has also been called 'crossover assurance'. On the other hand, the reduced probability of occurrence of one crossover in the vicinity of another has been termed 'crossover homeostasis'. Some authors have suggested that the underlying mechanism for these two phenomena is different, while others have argued that both are likely to originate from the same cause.

Positive crossover interference governs the patterns of inheritance of blocks of genes, the linkage groups. It is therefore of outstanding importance to get to grips with the underlying mechanism(s), not only for theoretical, genetic, reasons but also to facilitate the design of breeding experiments in plants and domestic animals as well as the development of personalized medicine and drug treatment. It goes without saying that numerous investigations have been undertaken to understand its origin.

**Figure 2:**
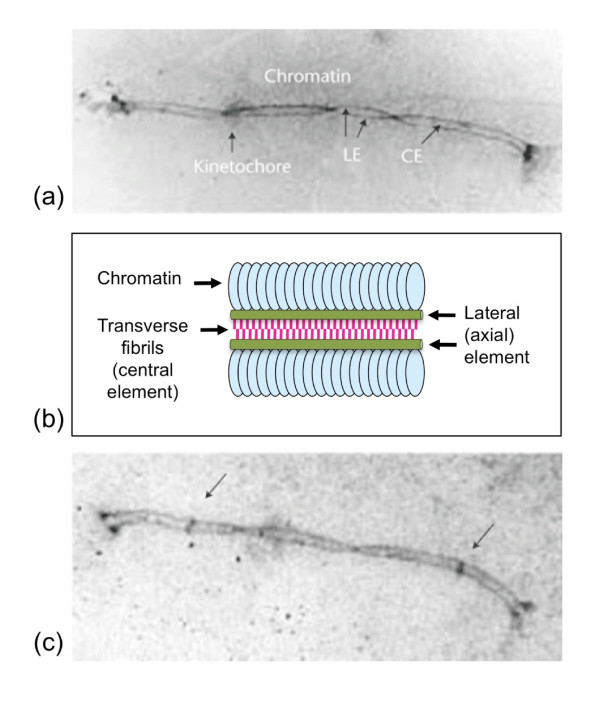
**The Synaptonemal Complex**. The Synaptonemal Complex (SC) is a meiosis-specific supra-molecular protein assembly that supports synapsis of homologs, crossover/chiasma formation and reciprocal recombination between sister chromatids at the Pachytene stage of Meiosis I. The chromatids of each homolog are held together by the Lateral Element (LE) consisting of cohesin proteins, formed already at the earlier Leptotene stage and then called the Axial Element [150]. The LE holds the two chromatids of each homolog tightly together until the onset of Anaphase I (see Fig 1). The central Element (CE) of the SC, made up of additional meiosis-specific proteins that hold the homologs together in a Velcro type of fashion, is required for maturation of early recombination events into crossovers/chiasmata [153]. (a) Electron-microscopy picture of the SC from a human male showing the Lateral Elements (LE) holding the two chromatids of each homolog together, the Central Element (CE), and the surrounding chromatin loops. Courtesy of N. Saadallah. (b) Schematic illustration showing the Lateral Elements (green), the Central Element, consisting of transverse fibrils (red), and the surrounding Chromatin (blue). (c) Electron-microscopy picture of the same bivalent as in (a) focused in such a way that the Late Recombination Nodules, corresponding to the crossovers/chiasmata are highlighted (arrows). The telomeres at each end, forming so-called attachment plaques, are associated with the nuclear membrane. Courtesy of N. Saadallah. Revised from [3].

The identification of crossover/chiasma interference has been based on (1) genetic recombination maps, more recently created primarily by tracing DNA markers along the length of individual chromosomes between parents and offspring, (2) chiasma maps illustrating the positioning of crossovers/chiasmata by light microscopy at the Diakinesis/Metaphase I stages of meiocytes, and (3) Late Recombination Nodules/MLH1 maps showing the positions of crossovers/chiasma formation at the earlier Pachytene stage of Meiosis I Prophase, using electron/immuno-fluorescence microscopy. At this stage of Meiosis I homologs are normally held together by a meiosis-specific proteinaceous structure, the so-called Synaptonemal Complex (SC) illustrated in Figure 2.

Remarkably, the basic underlying mechanism(s) for positive crossover/chiasma interference is/are still not understood, and this constitutes an outstanding biological enigma. A number of different models have been proposed, reviewed in [8-15]. In this paper I present a model for positive crossover/chiasma interference, based on the relative mechanical impact of oscillatory movements of homologous chromosome pairs during the Prophase stage of Meiosis I, induced respectively at the telomeres via the nuclear membrane and the centromeres via the kinetochores. In so doing I presume that both crossover assurance and crossover homeostasis are caused by the same basic mechanism.

I suggest that crossing-over and chiasma formation is facilitated by the proximity of parental homologs at the nodal regions of the waves thus created. I further propose that this model may adequately explain the salient features of crossover/chiasma patterns and interference, i.e. (1) there is normally at least one, obligate, crossover/chiasma per bivalent, (2) the number is correlated to bivalent length, (3) the positions are dependent on number, (4) the interference distances are on average longer over the centromere than along chromosome arms, and (5) there are significant changes in carriers of structural chromosome rearrangements.

Mathematical aspects of this and previously published interference models will be presented separately (Clocksin et al. in preparation).

## Results and Discussion

The only way in which it is possible to visualize crossover distribution along the length of all the individual chromosomes simultaneously is by cytogenetic analysis of meiocytes. Cytogenetic methods thus provide a means to determine directly the patterns of recombination both across the whole genome and at the chromosomal level, information that cannot readily be obtained in any other way [reviewed in [16-18]]. I will therefore here focus attention on the results illustrated by this type of investigation. As my special interest concerns the crossover picture of human chromosomes in relation to that in the mouse, my analysis will be biased to this effect. With reference to the COM model the parameters to consider include: (1) the mitotic karyotype, i.e. ranked length and centromere index of the chromosomes involved, (2) the specific bivalent/multivalent length and condensation/flexibility, dependent on the way this structure is positioned within the nucleus and the size of the respective meiocyte nuclei, (3) the frequency characteristics of the oscillatory movements at respectively the telomeres and the kinetochores.

I will in the following be looking at the cytogenetic information relevant to the understanding of the origin of crossover/chiasma interference separately as regards (1) Chiasmata at the Diakinesis/Metaphase I stage of Meiosis, (2) MLH1 foci at the Pachytene stage of Meiosis I Prophase, (3) Crossover patterns in mammals other than humans and mice, (4) Crossover patterns in other eukaryotes, (5) Telomere and kinetochore movements during Meiosis I.

### 1. Chiasmata at the Diakinesis/Metaphase I stage of Meiosis

**Figure 3:**
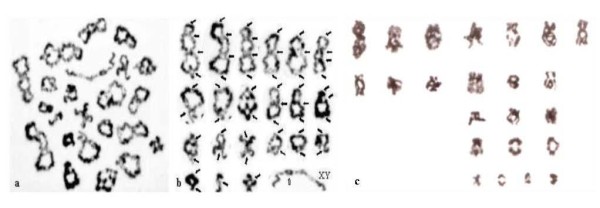
**Human spermatocyte and oocyte at the Metaphase I stage**. The chiasmata of the spermatocyte (a) have been highlighted (b). Note the difficulty in identifying the chiasmata in the oocyte (c) in comparison to those in the spermatocyte (a, b). Revised from [87] and [154].

Most information on the frequency and distribution of chiasmata along the lengths of individual human chromosomes has been obtained by microscopy analysis of spermatocytes at the Diakinesis/Metaphase I stage in testicular samples from adult males (Figure 3a, b). By comparison there is little corresponding information on the chiasma frequency distribution in the human female. One of the main reasons for this discrepancy is likely to be the access to the material for study.

**Figure 4:**
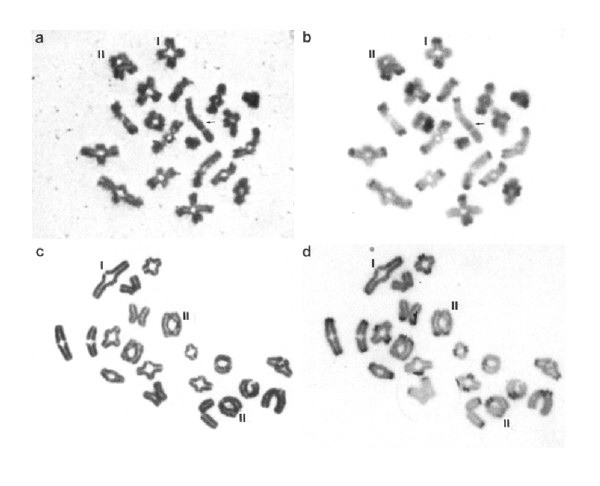
**The chiasma pattern in normal mouse chromosomes**. Mouse spermatocytes (top) and oocytes (bottom) at the Metaphase I stage from mice with normal karyotypes after block staining (a, c) followed by C-banding (b, d). The XY bivalent of the spermatocyte is arrowed. Note examples of mono-chiasmate (I) and di-chiasmate (II) bivalents. Reproduced from [20].

The Diakinesis/Metaphase I stage in oocytes takes place just before ovulation, usually with only a single oocyte in division at any one time. Also, the morphology of the chromosome pairs and the identification of the chiasmata at this oocyte stage (Figure 3c) are not as clear as that in spermatocytes (Figure 3a, b). This difference has precluded detailed information from being obtained on the chiasma frequency and distribution in human oocytes in comparison to spermatocytes. On the other hand, information on chiasma frequency and distribution in female mice has been obtained following short time *in vitro *culture of oocytes (Figure 4; see e.g. [19,20]). There is now also quite a lot of information on the crossover patterns in human and mouse spermatocytes and oocytes obtained by immuno-fluorescence analysis of MLH1 foci at the Pachytene stage of Meiosis I, as described in more detail in the following section.

#### 1.1 The chiasma patterns in males with a normal karyotype/spermatogenesis

The first detailed analysis of chiasmata in spermatocytes, obtained by testicular biopsies from human males with normal mitotic karyotypes and normal spermatogenesis, was performed in the 1970s [21-26]. In summary these studies demonstrate: (1) the occurrence of a so-called obligate chiasma, i.e. the fact that normally each chromosome pair (bivalent) undergoes at least one crossover, (2) a positive correlation between bivalent length and number of chiasmata, (3) the distribution of chiasmata being dependent on their numbers with a single chiasma often localized in the middle of the respective bivalents, in contrast to the situation in bivalents with higher number of chiasmata, showing a tendency for additional chiasmata to become placed nearer to the telomeres, (4) the interference distances being increasingly shorter with increasing number of chiasmata, (5) the interference distance on average being longer over the centromere in comparison to that along the lengths of individual chromosome arms, and (6) the pattern of interference being significantly changed in carriers of structural chromosome rearrangements. Further studies during the next few decades have substantiated these observations, and also demonstrated the existence of inter-individual variation in chiasma frequency and distribution between normal human males [27-31]. Similar observations have been made in mice [see e.g. [19,20,32,33]].

**Figure 5:**
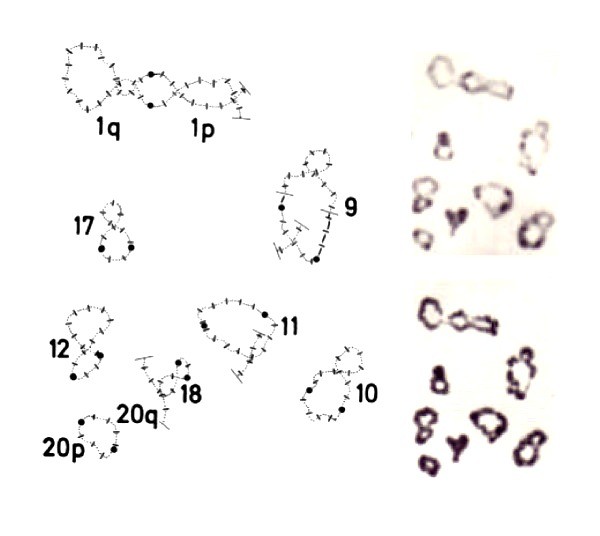
**Measurements of chiasma positions**. Spermatocytes are photographed, following consecutive triple staining with Quinacrine Mustard, Orcein (top right) and C-staining (bottom right) and drawings then made from the projected pictures at approximately 7000 times enlargement. Measurements are made of the chiasma positions in relation to the centromeres (left). Revised from [25].

**Figure 6:**
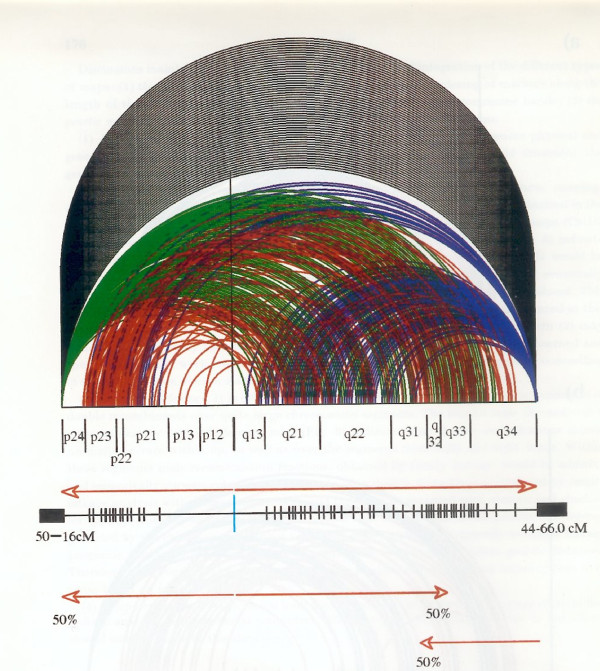
**Chiasma Interference Map (CHIM) of human chromosome 9**. The data are based on direct measurements (see Fig 5) in 366 spermatocytes from 10 normal human males. The × axis represents the chromosome and the vertical black line the centromere position. The figure illustrates the frequency of chiasmata along the length of the chromosome arms and the interference distances in each spermatocyte separately. Interference loops, which involve nearly the whole chromosome are black, the near telomere plus interstitial ones are green and blue respectively, dependent on whether the near terminal chiasma is located at 9p or 9q, while those which involve interstitial chiasmata only are red. The barcode diagram shows the chiasma-derived 1 cM genetic map calculated from the centromere towards the telomeres. Note the large pericentromeric gap. The near telomere gaps are artifacts due to any chiasmata within the width of the chromosome being recorded as strictly terminal. Revised from [31].

Measurements of chiasmata along the lengths of individual chromosomes (Figure 5) have allowed Chiasma Interference Maps (CHIMs) to be produced for each individual human and mouse chromosome, examples of which are shown in Figure 6, 7. Looking at these CHIMs it would appear that the frequency distribution of chiasmata is dependent on some specific features, located at the nuclear membrane as well as at the centromeres. One straight forward interpretation for these patterns is that this reflects oscillatory chromosome movements, taking place at the time of chiasma formation during Meiosis I Prophase. I would thus suggest that chiasmata are preferentially formed at the nodal regions of any such waves, created at the telomeres attached to the nuclear membrane and the kinetochores within the centromeres. In order to accommodate the interference distance spanning the centromere being longer than that within chromosome arms, I presume that the nuclear envelope/telomeric oscillatory movements are counteracted by those created at the centromeres/kinetochores, operating in both directions. I also envisage that once established the accumulation of crossover proteins (such as the MLH1 and MLH3) may clamp homologs, causing adjacent chromosome segments to splay, thus preventing additional adjacent crossovers to be formed at any nodal regions created by subsequent waves.

**Figure 7:**
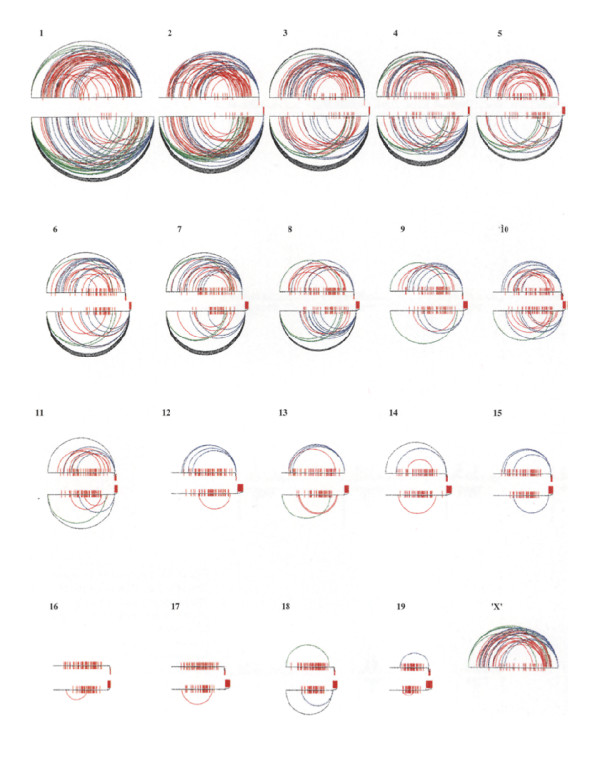
**Chiasma interference maps (CHIMs) of mouse chromosomes**. Chiama interference maps (CHIMs) for spermatocyte (upper) and oocyte (lower) chromosome rank sizes with the centromeric heterochromatin situated to the left. Single chiasmata are represented by vertical red bars, crossing the axis with distal clusters projected outside the axis. Multiple chiasmata within each bivalent are joined by loops, illustrating the chiasma interference patterns. Loops joining extreme proximal and interstitial chiasmata are shown in green, and those joining extreme distal and interstitial ones in blue. Loops joining extreme proximal and extreme distal chiasmata are black and those joining two interstitially located chiasmata are red. Note the wider distribution of interstitial chiasmata in comparison to the corresponding, near terminal ones in multiple chiasma bivalents. Revised from [20].

**Figure 8:**
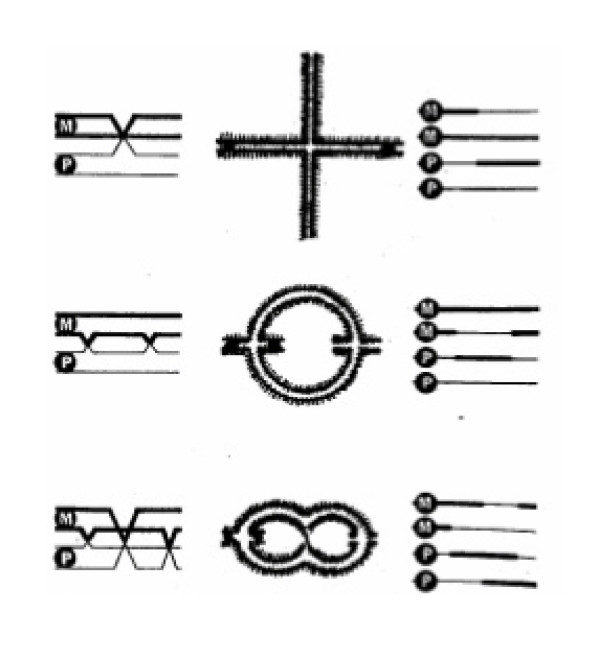
**The three types of chiasma formation in acrocentric chromosomes**. The drawing illustrates the crossover patterns/chiasma formation and reciprocal recombination between homologous chromatids in acrocentric chromosomes, which as regards singles and doubles are basically the same in mice and humans. Note that the different chromatids from the two homologs are randomly selected, i.e. there is no chromatid interference. A single chiasma is most often positioned either medially or more distal towards the telomere (top). Two chiasmata are located more near to the centrome and telomere (middle). Three chiasmata are located, respectively, adjacent the centromere, medially and adjacent to the telomere. Reproduced from [155].

It is in this context essential to note that it is not the centromere *per se *that inhibits chiasmata to be formed near its vicinity. This is obvious from the patterns seen in acrocentric chromosomes (Figure 3, 4, 7, 8; see e.g. [34,35]). The small human acrocentrics (21 and 22) usually harbor only a single interstitially or near terminally positioned chiasma, while the longer ones (13-15) have one, two or three chiasmata. Singles are positioned interstitially/medially, or near terminally. In double- and triple chiasma bivalents, however, the proximal chiasma is located adjacent to the centromere and the distal nearer to the telomere. This pattern is very similar to that in wild type/normal laboratory mice (Figure 4, 7; [19,20,33]). In accordance with the COM model I have interpreted this standardized pattern of chiasma frequency distribution in human and mouse acrocentrics to be the result of the conjoined oscillatory action of the telomeres and the kinetochores (via the heterochromatc short arm) being abutted to the nuclear membrane.

On a more general note it is also important to recognize that chiasmata are in fact already locked into their original positions at the transition between the Pachytene and Diplotene stage of Meiosis I. Thus, the original suggestion in 1929 by Darlington [36] as recently reiterated by e.g. de Boer et al. [37] that the frequent occurrence of near-terminal positions of chiasmata is due to their movement from their original interstitial positions (so-called chiasma terminalization) is a misconception [27,30,38-41].

**Figure 9:**
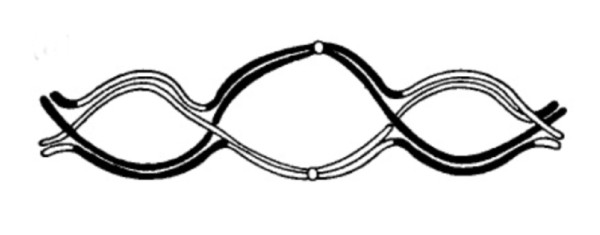
**Chiasma positions at the Diplotene stage as seen in 2D**. It would from this picture in 2D appear that chiasmata would have to move towards the ends of the chromosome (terminalize) in order for homologs to be able to separate at the following Anaphase I stage. In reality, however, any such movement is prohibited by the inter-chiasma loops being orientated perpendicular to each other. Reproduced from [30].

Looking at the bivalent in 2D it would seem necessary for chiasma terminalization to take place before homologs are able to separate (Figure 9). However, as soon as the telomeres are disconnected from the nuclear membrane in the transition between the Diplotene and Diakinesis stages, the chromosomes condense and at the same time they are transformed into 3D structures, where each interference/inter-chiasma segment is located perpendicular to the next. Chiasmata are thus bound to remain in their original positions as laid down at the Pachytene stage of Meiosis Prophase I. One mechanically favorable result of this 3D arrangement (similar to that in an ordinary metallic chain) is that any kinetochore-induced chromosome movement towards the opposite spindle poles at Anaphase I of Meiosis induces a separation of chromatids in adjacent interference loops.

It is also essential to appreciate that the large-scale preferential/optimal crossover placement/chiasma formation along the length of each individual bivalent as dictated by crossover/chiasma interference is not related to G-banding/chromomeres or any DNA specification but primarily dependent on chromosome morphology and bivalent length *per se *[42-46]. On the other hand, it is now well known that, at the DNA level, certain DNA sequences within these chromosome segments constitute so-called 'crossover hotspots'. Mammalian crossover hotspots, corresponding to initial DNA breaks are around 1-2 Kb long DNA segments that are separated by larger intervals with very low frequencies [47-52].

Only a small proportion (around 1 in 500 in the human male) of the specific DNA motif (recognized by the PRDM9 protein) within these 1-2 Kb long crossover hotspot are, however, selected for the final crossover and chiasma formation; and I am here discussing a model aimed at explaining the classical type of crossover/chiasma interference, involving many Mb of DNA. Thus, I am not addressing the mechanism(s) underlying any interference involved in the interaction between homologs, taking place as part and parcel of the complex molecular pathway leading up to final crossover/chiasma formation and reciprocal recombination. For a detailed analysis of these factors in relation to previous models of crossover interference readers are referred to the recent presentations in [12-15].

#### 1.2 The chiasma patterns in carriers of structural chromosome rearrangements

Analysis of chiasma interference has also been performed in human male carriers of structural chromosome rearrangements. Most attention has focused on reciprocal translocations, where in the majority of spermatocytes at the Diakinesis/Metaphase I stage a quadrivalent configuration has been seen. The chiasma frequency distribution has been studied in a relatively large number of such human male carriers (see e.g. [23,26,53-75]).

**Figure 10:**
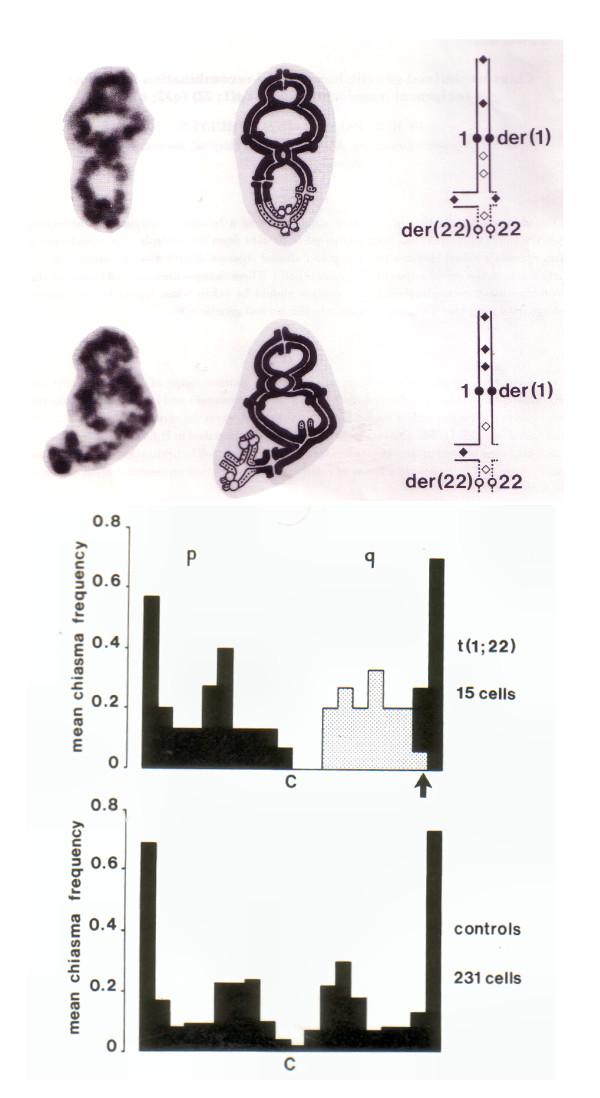
**The chiasma pattern in a human male reciprocal 1;22 translocation carrier**. Pictures of translocation quadrivalents at the Diakinesis/Metaphase I stage in spermatocytes from a carrier of a reciprocal 1; 22 translocation with the corresponding drawings showing the interpretation of the chiasma patterns (top). Note the high frequency and different distribution of chiasmata within the interstitial segment between the centromere and the breakpoint. The translocation carrier shows a raised chiasma frequency and altered chiasma distribution in chromosome 1, particularly in the region adjacent to the breakpoint (grey staples) in comparison to six controls with normal karyotypes (bottom). Reproduced from [64].

In the reciprocal translocation carriers where the chiasma frequency distribution has been analyzed in detail, the most striking deviation from the situation in human males with normal karyotypes is a significant increase in the frequency of chiasmata localized within the interstitial segment, i.e. the chromosome segment positioned in between the breakpoint and the centromere (Figure 10; see also Figure 8 in [65]). This is true even when the interstitial segment is very short. In stark contrast to the normal situation in non-acrocentric chromosomes there is then a tendency for chiasmata to occupy positions near to/adjacent to the centromere, as well as a substantial reduction in the crossover/interference distance over the centromere. A similar tendency for an increased frequency of chiasmata within the interstitial segment has been seen in reciprocal translocations in mice [76,77].

Under the COM model I would suggest that the explanation for this deviant pattern of chiasma frequency distribution in the quadrivalent in comparison to the normal is the change in the mechanics of the waves induced by the oscillatory movements of the telomeres/the nuclear membrane in relation to those of the kinetochores/centromeres. First of all, the quadrivalent has to accommodate waves originating from four different places along the nuclear membrane travelling to its centre. Second, the situation is further complicated by the quadrivalent having two rather than one duplex kinetochore and the potential associated alteration in effect on the nodal regions of the chromosomal waves caused by their oscillation.

**Figure 11:**
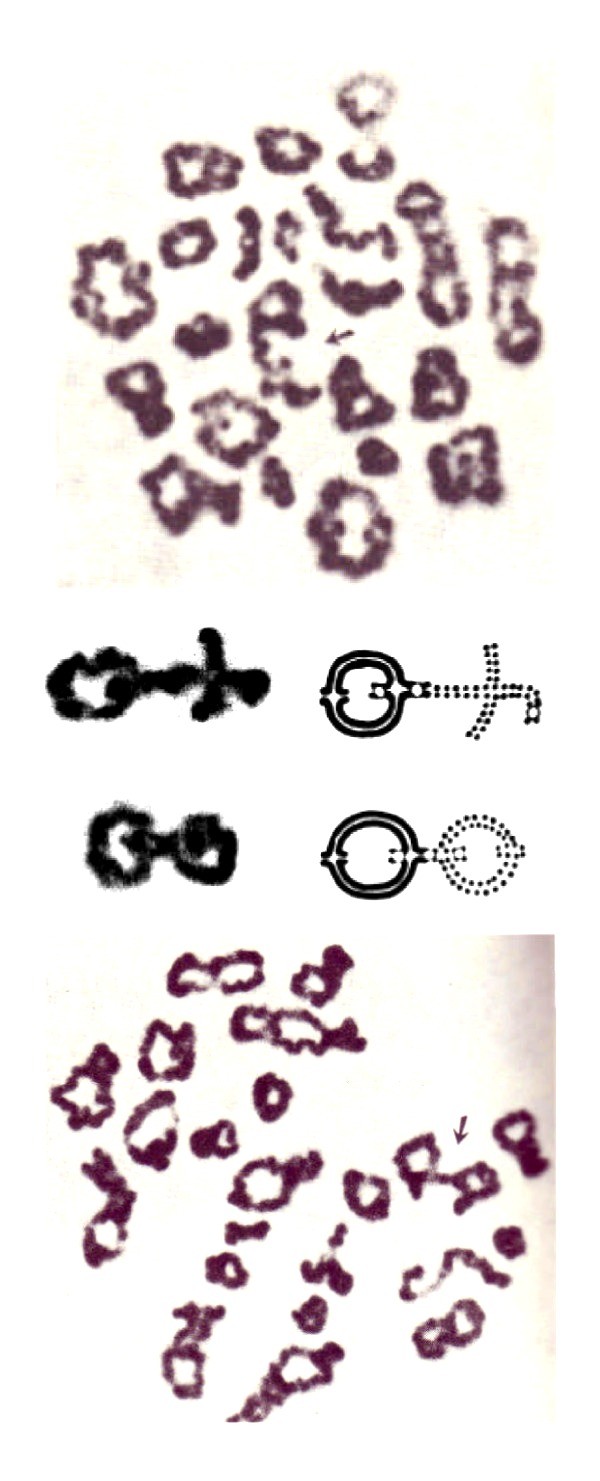
**Chiasma pattern in a carrier of a 13; 14 Robertsonian translocation**. Trivalents containing either two chiasmata on one arm together with two chiasmata on the other (arrow, top) or two chiasmata on both arms (arrow, bottom) and two additional examples (cut outs, middle). Note the dicentric nature of this metacentric derivative 13; 14 Robertsonian translocation, as illustrated by the drawings (middle). These chiasma patterns of the trivalents in this carrier are similar to those of the individual chromosomes 13 and 14 in human males with normal karyotypes. Revised from [26] and [155].

The chiasma patterns seen in spermatocytes from human Robertsonian translocations are of special interest, demonstrating quite clearly the influence of karyotype and chromosome morphology. The chromosome arms of the trivalents in the common 13; 14 and 14; 21 translocations (formed by the two normal together with the translocation chromosome) show the same pattern as that in the normal situation of the respective acrocentrics (Figure 11; [26,54]). This apparently normal chiasma pattern within individual chromosome arms in trivalents of the human heterologous Robertsonian translocations is also seen in the corresponding mouse Robertsonian fusions [78,79]. This pattern is that expected on the COM model, as these trivalents are likely to be positioned in the same way as their corresponding normal bivalents within the meiotic cell nuclei, with the normal telomere and centromere movements thus retained.

**Figure 12:**
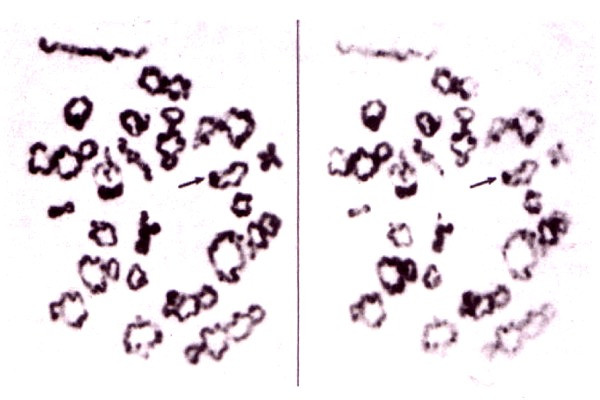
**Chiasma pattern in a carrier of a 14; 14 Robertsonian translocation**. Spermatocyte at the Diakinesis/Metaphase I from a human male carrier of a 14; 14 Robertsonian translocation, stained with C-banding (left) and orcein (right) where the univalent 14; 14 is arrowed. Note the parallel centromeres, showing that synapsis has occurred between chromatids from the two different homologs, and the occurrence of a single chiasma very near the telomere. Reproduced from [23].

In stark contrast, the univalent in the unique case of a human 14; 14 metacentric Robertsonian translocation invariably forms a ring with a single distal chiasma, different to the rings of the normal chromosome 14 bivalent, having two chiasmata, one at each end (Figure 12, cc Figure 7, 8). The derivative 14; 14 chromosome is dicentric, where the proximal telomeres have been lost. According to the COM model the single distal/near telomeric chiasma in the Diakinesis/Metahase I univalent is likely to be due to the effect of the oscillatory movements induced by the dual kinetochores counteracting those originating at the dual distal telomeres, both attached to the nuclear membrane.

Yet again, in stark contrast, the chiasma patterns in the mouse homologous Robertsonian translocations seemingly correspond to that expected on the basis of the oscillatory movements, similar to those in the middle-sized human metacentric/submetacentric chromosomes with either two or three chiasmata, forming rings or figures of eight (cc Figure 1 in [33] and Figure 3a here).

Finally, as regards structural heterozygotes, inversion carriers present an especially complex case, associated with the particular problems encountered in the pairing and synapsis of parental homologs, which is a pre-requisite for their interaction in the process of crossing over/chiasma formation [review in [80]]. In inversion heterozygotes involving a short chromosome segment there is a possibility of its elimination from synapsis by looping out, leading to a corresponding reduction in homologous crossing-over in this particular chromosome segment [60]. From a mechanical point of view the situation in carriers of larger interstitial inversions is even more complicated, both as regards initial homologous synapsis, so called non-allelic homologous synapsis and synaptic adjustment, identified by detailed EM analysis [80-82]. Further studies on the patterns of crossover/chiasma formation are required before any firm conclusion can be drawn as regards the interpretation of their origin in relation to the COM model.

Intriguingly, in carriers of a double inversion of chromosome 1 in mice, a reduction of chiasmata has been seen in single heterozygotes but an increase in the double heterozygotes, the latter associated with a reduced strength of interference [83]. In order to provide an adequate explanation in particular for the apparent decrease in strength of interference in the double hetrozygote it would be helpful to obtain additional information on the relation between synapsis [84] and crossovers by way of MLH1 analysis at the Pachytene stage (see section 2). The same holds true as regards a double heterochromatic insertion in the middle of the mouse chromosome 1 [85]. Both such heterozygotes and homozygotes show an increase in chiasma frequency with the normally medial chiasma replaced by one proximal together with one distal/pro-terminal. It may seem likely that this is somehow related to the well known prevention of chiasma formation within heterochromatic segments. Perhaps the expected looping out of the two heterochromatic blocks includes the interstitial euchromatic section (see e.g. [86]), thereby preventing crossovers within this segment?

#### 1.3 The chiasma patterns in human males with non-obstructive azoospermia

**Figure 13:**
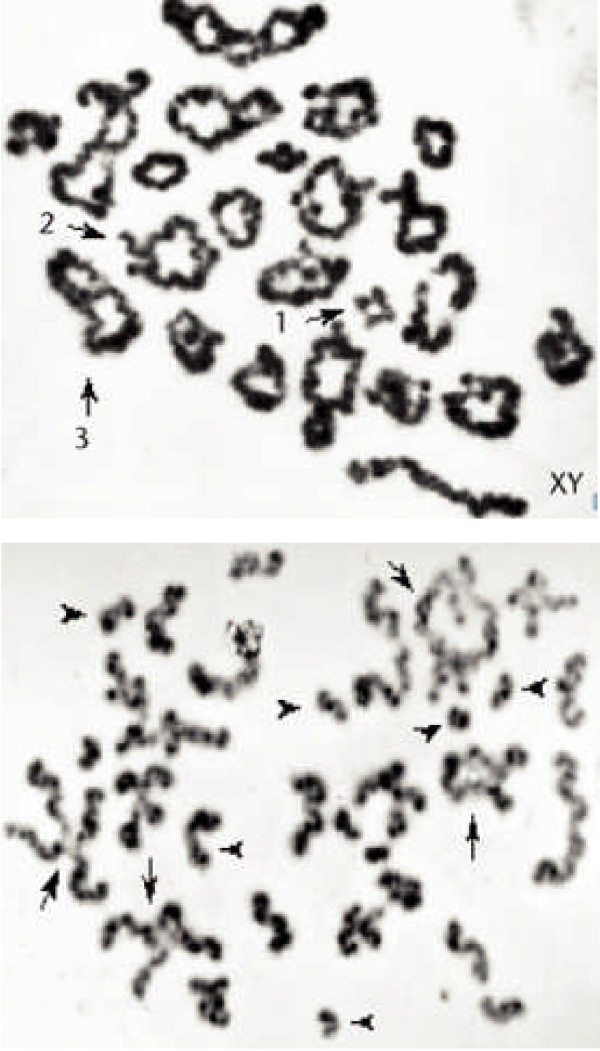
**Positional control of chiasma formation**. Spermatocytes at the Diakinesis/Metaphase I stage from a normally fertile human male with normal mitotic karyotype (top) in comparison to that in a male with non-obstructive azoospermia (bottom). Note the extremely low number of chiasmata in the spermatocyte from the azoospermic male; some chromosomes lack chiasmata altogether (I) while two relatively large bivalents show a single chiasma a medial position (arrowed, left). There are also a number of univalents (arrow heads) as well as two bivalents with two chiasmata (arrowed, right). Reproduced from [156].

Chiasma analysis at the Diakinesis/Metaphase I stage in a number of studies on spermatocytes from testicular biopsy samples of men suffering from reduced fertility associated with non-obstructive oligo-azoospermia has shown that some have disturbances in chiasma formation [87-90]. In a first comprehensive study of 50 men with this condition [87] the majority (n = 41) was found to have a normal progression of spermatogenesis and a normal, or nearly normal, chiasma pattern. Among the remaining 9/50 cases, 7/50 showed spermatogenic arrest already at the Pachytene stage of Meiosis I, and no information on crossover/chiasma formation could at the time be obtained. In two exceptional cases the majority of parental homologs in spermatocytes reaching the Diakineses/Metaphase I were unpaired. Some of these spermatocytes did, however, show the occasional apparently normally paired bivalents, illustrating the notion of positional control of chiasma formation. Even in this aberrant situation a single chiasma in a large bivalent occupied a medial/central position (Figure 13). The same has more recently been seen in some oligo-azoospermic men, where the crossover pattern has been studied by MLH1 focus analysis of spermatocytes at the Pachytene stage of Meiosis Prophase I, as described in the following section.

### 2. MLH1 foci at the Pachytene Stage of Meiosis I Prophase

**Figure 14:**
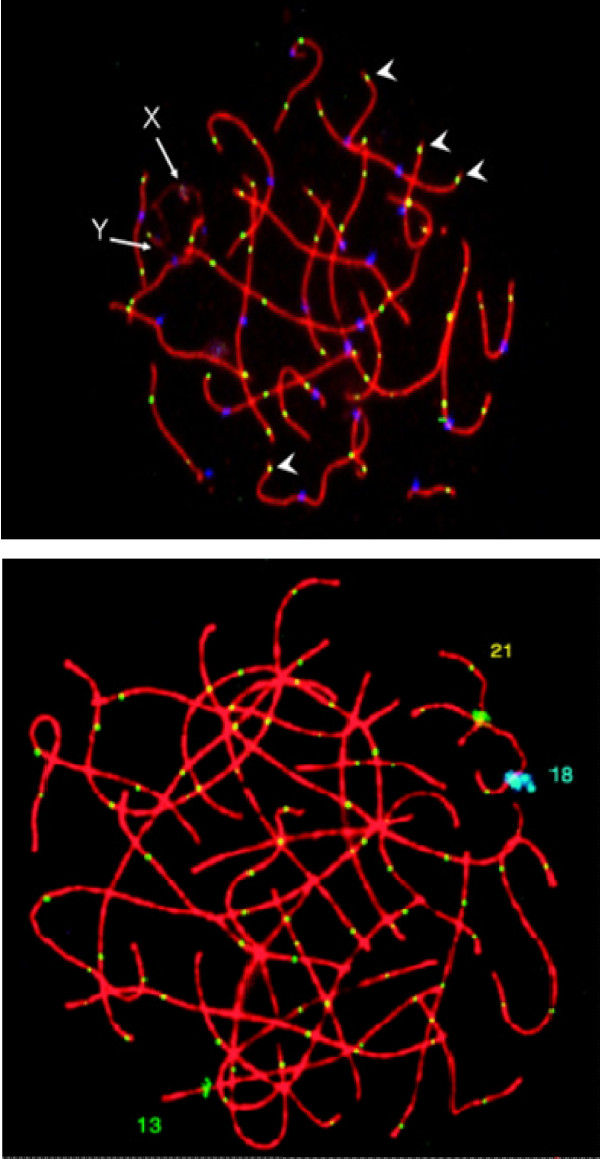
**MLH1 foci along the Synaptonemal Complexes (SCs) in human males and females**. The spermatocyte (top) and oocyte (bottom) have been stained using antibodies against SCP3 (red), MLH1 (yellow) and, in the spermatocyte, the kinetochore (blue). Homologs 21, 13 and 18 have been identified in the oocyte, using Fluorescence in situ hybridisation (FISH) with centromere-specific probes. There are obvious differences between the spermatocyte and oocyte: the SCs are much longer in the oocyte; there are more MLH1 foci in the oocyte; MLH1 foci tend to be positioned closer to the telomeres in the spermatocyte (arrow heads). Reproduced from [86] and revised from [94].

As would be expected from the correspondence between the positions of MLH1 foci analyzed at the Pachytene stage of Meiosis I and chiasmata at the later Diplotene/Diakinesis/Metaphase I stage in Ocadaic Acid stimulated spermatocytes of mice and men ([91] and Khazanehdari and Hultén (unpubl. obs.)) the deduced crossover patterns are largely congruent. One advantage of the MLH1 approach concerns the number of spermatocytes that can be readily analyzed, dependent on the much longer duration of the Pachytene stage of Meiosis I in relation to that at the short Diakinesis/Metaphase I stage. Another advantage is that the MLH1 analysis can be performed on equally large populations of human oocytes at the Pachytene stage of Meiosis I obtained from fetal ovarian biopsies. The possibility of obtaining information on the crossover patterns in both human males and females has in particular allowed a detailed comparison to be made as regards any sex difference in crossover/chiasma interference distances. Figure 14 shows the typical pattern of MLH1 foci in a spermatocyte in comparison to that in an oocyte.

#### 2.1 MLH1 focus analysis in human males with normal mitotic karyotypes

Analysis of MLH1 foci at the Pachytene stage of Meiosis I in spermatocytes from a range of human males with normal mitotic karyotypes and normal spermatogenesis has by and large produced a very similar picture of crossover interference to that obtained by chiasma analysis at the Diakinesis/Metaphase I stage (cc Figure 3a, b and 14; [42,92-102]). This is also the case in mouse spermatocytes [103,104].

The mathematical model on crossover interference by Falque et al. [105], based on the MLH1 data by Froenicke et al. [104], will be discussed in conjunction with our own mathematical version of the COM model to be presented separately (Clocksin et al. in preparation).

#### 2.2 MLH1 focus analysis of carriers of structural chromosome rearrangements

MLH1 foci have also recently been investigated in a number of human male carriers of reciprocal translocations with normal spermatogenesis [104-107]. This work provides valuable new information on synaptic problems with respect to crossover frequency distribution. There is, on the other hand, no substantial new information as regards crossover interference. The same holds true for Robertsonian translocations and inversions in mice [[96,110], review in [111]].

#### 2.3 MLH1 focus analysis in men with non-obstructive azoospermia

By comparison to reports describing the chiasma patterns at the Diakinesis/Metaphase I stage in men with non-obstructive oligo-azoospermia discussed above there are a relatively large number of human males, where MLH1 focus analysis of spermatocytes at the Pachytene stage of Meiosis Prophase I has been used to highlight crossover frequency distribution [see e.g. [95,98,100,108,109,112-116]]). Again, the picture revealed is very similar with the majority having some reduction in deducted crossover frequency. Figure 3c of Gonsalves et al. [95] demonstrates the positional effect with the medial/central position of a single MLH1 focus in a large chromosome (cc the position of the chiasma in Figure 13 here).

#### 2.4 MLH1 focus analysis of human oocytes at the Pachytene stage

As illustrated in Figure 14, the numbers of crossovers estimated from MLH1 foci, is higher in oocytes than in spermatocytes. Initial MLH1 analysis [92,94] has suggested an average of approximately 70 crossovers per oocyte with a larger inter-cell variability (range 40-100) in comparison to around 50 in spermatocytes (range 41-59). Subsequent studies have confirmed the occurrence of a large variation in crossover frequency between individual oocytes within subjects and also indicated a higher inter-individual variability [117,118]. The higher rate of recombination in oocytes is most probably related to the considerable difference in chromosome length at the Pachytene stage of Meiosis Prophase I [42,44]. Thus, the human female genome has a longer physical platform for establishment of crossing-over/chiasmata/reciprocal recombination than the male (Table two in [94]). Both the larger variation in crossover frequency between individual oocytes and the higher inter-individual variation is likely to be due to larger differences in oocyte nuclear size in comparison to that in spermatocytes. It should be added, on the other hand, that there is, by measurement of the meiosis-specific chromosome pairing structures, the Synaptonemal Complexes (SCs), evidence to suggest that the strength of interference is similar in both sexes in terms of actual physical distance between crossovers/chiasmata. Thus, the rate of recombination per unit length of SC is relatively constant in the two sexes, when the influence of the "obligate chiasma" is discounted [44].

Not only do the two sexes show a significant variation in recombination frequency, but they also display some differences in distribution [94,117,118]. In spermatocytes, the MLH1 foci/chiasmata are often located very close to the ends of the chromosomal axes. In oocytes, MLH1 foci are located more interstitially (away from chromosome ends) and only very rarely positioned so near to telomeric segments as in spermatocytes (Figure 14). On the COM model I have interpreted this sex difference in crossover numbers as well as positioning to be related to the effect of the telomeric and kinetochore oscillatory movements of the longer and thinner, and therefore more flexible, female chromosome pairs.

### 3. MLH1 Foci Patterns in Mammals other than Humans and Mice

Additional information on the crossover frequency distribution has more recently been obtained on normal spermatocytes (and in a few cases also oocytes) in a number of different mammalian species, i.e. by analysis of MLH1 foci in domestic animals [review in [119]]as well as cat [120], common shrew [121], dog [122], American mink [123], Rhesus [124] and silver fox [125]. By and large these studies reiterate the notion that the patterns of distribution of crossovers along the length of individual bivalents are very similar, to a large extent being dependent on chromosome morphology, as reflected by bivalent length and centromere/kinetochore position. It is noteworthy, however, that unusually short intra-arm interference distances have been identified in cat spermatocytes [120]. The reason for this exceptional behavior is not known, and requires further study. In the context of optimal mechanical stability, facilitating regular segregation of parental homologs at the Anaphase I stage, I would presume these dual crossovers would function in the same way as a single chiasma.

### 4. Crossover Patterns in other Eukaryotes

Investigation of crossover patterns in a wide range of eukaryotes indicates that crossover/chiasma interference is a characteristic feature in most. One extreme example of interference is seen in the nematode *Caenorhabditis elegans*, where all bivalents irrespective of their size have a single chiasma localized distally. The multiple sites of recombination initiation are then resolved into a single crossover, with the diffuse (holocentric) kinetic activity that extends along the length of the mitotic chromosomes being reduced to the single telomeric end of each meiotic chromosome, via direct insertion of the microtubules into the chromatin [review in [126,127]].

The only two known exceptions to the general rule of positive crossover interference in eukaryotes concern the fission yeast, *Saccharomyces pombe *together with the fungus, *Aspergillus nidulans*. In both of these organisms crossovers are randomly distributed along the length of individual bivalents, and both lack the meiosis-specific chromosome pairing structure, the so-called Synaptonemal Complex [[128,129], see also [130]]. Much attention has been paid to the underlying reason for this random distribution of crossovers in fission yeast, involving the clustering of telomeres in a restricted area of the nuclear membrane (bouquet) and the movement of the nucleus back and forth in the cell by a so-called horsetail formation [129-134].

### 5. Meiotic Telomere and Kinetochore Movements

Interest has recently focused on oscillatory movements of the telomeres during Prophase of Meiosis I, when homologous parental chromosomes align and pair intimately (synapse) to allow crossing-over between non-sister chromatids to take place [see e.g. [135-141]]. No conclusion has, however, been reached as regards their exact role(s) with respect to the patterns of crossover/chiasma frequency and distribution. Most recently it has been suggested that these movements may eliminate unwanted inter-chromosomal associations or entanglements that have arisen as part and parcel of the homolog pairing process [141].

Much less attention has been paid to any corresponding movements of the kinetochores at the Prophase stage of Meiosis I. Thus, information on meiotic kinetochore movements *per se *is currently restricted to that obtained at the later Metaphase I to Anaphase I transition [142-145].

With reference to the COM model I would be specially interested in further investigation of telomere and kinetochore movements at the Pachytene stage of Meiosis I in organisms with large chromosomes (such as maize, locusts and grasshoppers, mice and humans) using approaches similar to the ones already performed on human chromosomes at the mitotic Metaphase stage [see e.g. [146]]. It would also be helpful to get information on the behavior of kinesin proteins [review in [147]] and other potentially relevant proteins such as *Sgo1 *suggested to act at sister kinetochores to promote their bi-orientation in *Saccharomyces cerevisiae *[148], *klp3A *where *Drosophila *mutants show abnormal crossover distribution [149], TEL1 proposed to be involved in the regulation of interference [150] and the MCAK protein, recently found to be associated with chiasmata at the prometaphase stage in mouse oocytes [151].

## Conclusions and Perspectives

I have here described a model for the origin of the meiotic crossover patterns shared between most eukaryotic organisms. I have suggested that the patterns seen, with special reference to the non random distribution and the crossover/chiasma interference is related to the oscillatory movements of the telomeres attached to the nuclear membrane and the kinetochores within the centromeres. Thus, I have presumed that these oscillatory movements, taking place at the Prophase stage of Meiosis I, lead to waves of physical interaction between the homologous chromosomes, with the highest chance of final crossovers/chiasma formation/reciprocal recombination being restricted to the chromosome segments corresponding to the nodal regions of the waves thus created.

One advantage of this type of purely mechanical/physical model for the origin of crossover interference is that it may now be tested in mechanical/mathematical experiments using any string replica of the homologous chromosomes at the Prophase stage of Meiosis I. The parameters to vary in this type of experiment would include: (1) the mitotic karyotype, i.e. ranked length and centromere index of the chromosomes involved, (2) the specific bivalent/multivalent length and flexibility, dependent on the way this structure is positioned within the nucleus and the size of the respective meiocyte nuclei, (3) the frequency characteristics of the oscillatory movements at respectively the telomeres and the kinetochores.

Should it turn out that the oscillatory movements that I have postulated do not adequately explain the crossover frequency distributions observed, then it will be essential to explore in particular what other characteristics of the centromeres/kinetochores that may underlie the increased interference distance over the centromere and the variation induced by structural chromosome rearrangements in comparison to the normal karyotype. I would be especially interested in obtaining further information on the potential impact of the differential mass of the centromere/kinetochore and the 3D spatial orientation of the chromosomes within the meiocyte nuclei, which likely will influence the progression of the waves I have hypothesized regulate the patterns of crossover/chiasma frequency and distribution along the length of individual chromosome pairs. I envisage that it might in fact be possible to modify and possibly simplify the COM model, based on the results of such mechanical/mathematical analysis. Perhaps the specific characteristics of the centromere may mean that it is not necessary to imply any oscillatory movements induced by the kintechores, and the patterns seen could be explained by waves induced by the telomeres alone?

Either way, I do nourish a hope that we will within the next few years have reached a full understanding of the origin of the phenomenon of crossover interference, so that we may celebrate the centenary since its first discovery, by Sturtevant [4] and Muller [5].

## Conflicts of interests

The author declares that she has no competing interests.
